# Deep brain stimulation for obsessive compulsive disorder leads to symptom changes of comorbid irritable bowel syndrome

**DOI:** 10.3389/fpsyt.2025.1545318

**Published:** 2025-03-05

**Authors:** Mohamed A. Abdelnaim, Tobias Hebel, Verena Lang-Hambauer, Juergen Schlaier, Berthold Langguth, Andreas Reissmann

**Affiliations:** ^1^ Department of Psychiatry and Psychotherapy, University Regensburg, Regensburg, Germany; ^2^ Center for Deep Brain Stimulation, University Regensburg, Regensburg, Germany; ^3^ Clinic and Policlinic for Psychiatry and Psychotherapy, Mainkofen, Germany; ^4^ Department of Neurosurgery, University Regensburg, Regensburg, Germany

**Keywords:** DBS, IBS, deep brain stimulation, irritable bowel syndrome, OCD

## Abstract

**Introduction:**

Irritable bowel syndrome (IBS) is a common condition characterized by abdominal pain and altered bowel habits, affecting around 11% of individuals globally. It is linked to dysregulation of the brain-gut axis, with altered activity and connectivity in various brain regions. IBS patients often have psychiatric comorbidities like anxiety, or obsessive-compulsive disorder (OCD). Deep brain stimulation (DBS) is an established treatment option for severe, therapy-refractory OCD. It has been suggested that DBS for OCD could also have a beneficial effect on accompanying IBS-symptoms.

**Methods and patients:**

Nine patients with treatment-refractory OCD who underwent DBS in the bed nucleus striae terminalis (BNST) have been included in this study (4 males, 5 females, mean age: 39.1 ± 11.5 years). Patients were examined with the Gastrointestinal Symptom Rating Scale for Irritable Bowel Syndrome (GSRS-IBS) as well as the Yale-Brown Obsessive Compulsive Scale (Y-BOCS) both before the beginning of DBS as well as throughout several follow-up visits for 12 months following the start of DBS.

**Results:**

Three patients displayed clinically relevant levels of IBS-symptoms at baseline (GSRS-IBS scores at or beyond 32). All of those three patients showed a reduction of the GSRS-IBS score at the last follow-up (12-40%). For the other 6 patients, 5 of them showed also a reduction of the GSRS-IBS compared to the score at baseline. The mean score for all patients showed a descriptive trend toward score reduction throughout the study period and until the last follow up visit after 12 months. The mean Y-BOCS decreased from 31.11 at baseline to 16.50 at the last follow-up. Out of the 9 patients, 7 (78%) were considered responders with Y-BOCS scores decreasing between 37% to 74%. Moderate-to-large correlations between both scales could be observed at both the 9-month and the 12-month follow-up visit. However, none of these associations was statistically significant.

**Conclusion:**

In this study, we found alleviation of IBS symptoms after DBS of the BNST, along with improvement in OCD symptoms. Future research using larger sample sizes should address whether the reductions are tied to the improvement of OCD symptoms or if DBS exerts positive effects on IBS independently of OCD symptoms.

## Introduction

With a global prevalence of 11% (1), irritable bowel syndrome (IBS) is a very common condition. It is characterized by abdominal pain or discomfort with altered bowel habits such as constipation, diarrhea, or both. but without pathological alterations in bowel tissue (2, 3). Many affected individuals can control their symptoms by managing diet, lifestyle and stress, whereas others are substantially impaired in their quality of life (4, 5).

Despite being identified more than 150 years ago, IBS continues to pose a clinical challenge with only limited treatment options (6, 7). According to the biopsychosocial model, IBS symptoms arise from the interplay of psychological, behavioral, sociocultural, and environmental factors, but the exact pathophysiological mechanisms remain incompletely understood (8–12).

There is increasing evidence that IBS is related to abnormal processing of internal pain signals, leading to changes in visceral sensitivity (13). Both central and peripheral mechanisms have been proposed to play a role in the emergence of pain symptoms (14), with multiple studies linking IBS to a dysregulation of the brain-gut axis, in which an imbalance can manifest as either sensory changes in the peripheral nervous system or disruptions in central processing (15–18). The term “brain-gut axis” subsumes bidirectional communication between the gut and the brain, both at rest and during stimuli such as postprandial states or luminal distension, regulating motions, reflexes, and sensory perceptions in the gastrointestinal tract. These ongoing complex interactions between the gastrointestinal tract and the central nervous system are essential for maintaining homeostasis and regulating gastrointestinal physiology (16).

Neuroimaging studies of patients with IBS have shown increased activation in various brain regions including anterior cingulate cortex, mid cingulate cortex, amygdala, anterior insula, posterior insula and prefrontal cortex (19). In individuals with IBS, these brain regions are linked to aberrant emotional arousal, intrinsic pain regulation, gastrointestinal hyperactivity and regulation of the hypothalamic-pituitary-adrenal (HPA) axis (20–23).

According to recent studies, patients with IBS are frequently suffering from psychiatric comorbidities like anxiety, obsessive-compulsive disorder (OCD) and depression (24–27). Among patients with functional bowel disorders, obsessive-compulsive disorder (OCD) is the second most prevalent psychiatric comorbidity, occurring in approximately 20% of cases (28, 29). For patients with OCD, similarly, the prevalence of gastrointestinal disorders is notably high, ranging from 14,9% (30), 16% (31) up to about 35% (32). Furthermore, patients with OCD are nearly twice as likely to report constipation of medically unexplained or mental origins (33). The co-occurrence and high prevalence of gastrointestinal symptoms should be thus a significant therapeutic consideration in the treatment of OCD patients, and vice versa (34).

OCD is a very debilitating disease, with approximately 40–60% of patients achieving only partial recovery with standard therapies, while around 10% of patients with OCD exhibit chronic, severe, and refractory illness, resulting in considerable functional impairments (35–37). Deep brain stimulation (DBS) represents a treatment option for severe, therapy-refractory OCD. In DBS, electrodes are stereotactically implanted in designated brain regions. Electrical impulses generated by a battery-driven stimulator situated beneath the skin of the upper chest are transmitted through these electrodes in order to affect brain activity in the targeted area(s). This procedure has become an established treatment option for patients with Parkinson’s disease, dystonia, tremor and other movement disorders. DBS has also been explored for the treatment of various psychiatric disorders with best evidence being available for the treatment of OCD (38). In 2009, the U.S. Food and Drug Administration (FDA) approved DBS for treatment-refractory OCD as a humanitarian device exemption (HDE H050003) (39). For the treatment of OCD, various targets were investigated including the anterior limb of internal capsule (ALIC) (40–42), the bed nucleus striae terminalis (BNST) (43), the ventral capsule/ventral striatum (VC/VS) (44–46), the nucleus accumbens (NA) (47–49), and the nucleus subthalamicus (STN) (50), with most of them reporting statistically significant effects (51, 52). This can be explained by the fact that many of the target areas are located in close proximity to each other or are functionally connected respectively. In a case report, it has been reported that DBS targeting the anterior limb of the internal capsule (ALIC) in a 55-year-old female patient with both OCD and IBS led to a substantial and reproducible reduction in IBS symptoms. This improvement was dependent on specific stimulation parameters and was not directly associated with changes in OCD symptoms (53).

Here we aimed to further explore effects of DBS in OCD patients on IBS symptoms. For this purpose, we analyzed IBS symptoms in our patients with treatment-refractory OCD who received DBS in the BNST (54). To do so, we investigated whether DBS in the BNST in patients suffering from OCD would lead to decreases both in measures of OCD as well as IBS symptom severity. Additionally, we looked at the correlation between the two symptom domains across the study period in order to get a deeper understanding of the potential temporal dynamics between symptom domains. Hence, we report of 9 patients, who underwent DBS in the BNST for their OCD between January 2021 and 2023 at the multidisciplinary center of deep brain stimulation at the University of Regensburg, Germany. Please note that the results of the same assessment instrument (e.g. Y-BOCS) may differ compared to the first study (54), as not all patients who were presented in the first study are also presented in the current one.

## Methods and patients

All patients have provided written informed consent to this study, which was approved by the ethic committee of the University of Regensburg (ethic vote: 21-2707-104). Potential candidates for DBS were screened for their eligibility first at the outpatient clinic of the department of psychiatry and psychotherapy and then at the outpatient clinic of the department of neurosurgery. The inclusion process consisted of multiple screening visits to confirm the OCD diagnosis, to check all available health records and to get a detailed summary of previous treatment trials, as well as to collect information on the patient’s psychosocial history and overall functioning.

The two main inclusion criteria were treatment resistance and disease severity. We defined treatment resistance as non-response to adequate trials with a maximum tolerated dose of at least two different serotonin reuptake inhibitors (SSRI) and one trial with clomipramine or augmentation with an antipsychotic (risperidone or aripiprazole) as well as non-response to cognitive-behavioral therapy (CBT) for at least one year (>50 sessions), including exposure therapy and *n*on-response to an adequate multi-professional treatment procedure (e.g., inpatient clinic with different therapy modalities). Regarding severity, we considered overall impairment in social, occupational functioning and patient’s normal routine.

Patients who met the criteria for DBS during the psychiatric assessment were referred to the neurosurgery department for evaluation of their surgical eligibility. Patients were provided with comprehensive information regarding the surgical procedure. All patients provided informed written consent for the surgical procedure, and the operation was conducted only after a minimum deliberation period of 60 days.

### Surgery

Preoperative MR imaging was conducted two days before the procedure using a 3T SIEMENS Magnetom Skyra scanner, with patients under general anesthesia throughout the imaging to prevent movement artifacts in preparation for DBS surgery. Sagittal T1 and axial and sagittal T2 images aligned with the intercommisural plane were obtained for trajectory planning, along with T1 images enhanced with a double dose of Gadolinium to delineate essential blood arteries and minimize the risk of hemorrhage during the insertion of stylets and DBS electrodes. On the day of surgery, a preoperative CT scan, utilizing a stereotactic frame affixed to the patient’s head (CRW, Integra Radionics, Burlington, USA), was acquired from a SIEMENS Somatom Definition Flash scanner and served as the reference for surgical planning. Trajectories avoiding relevant blood vessels, sulci and crucial neurological structures were defined using iPlanNet 3.0 (BRAINLAB, Munich, Germany) with targets in the bed nucleus striae terminalis (BNST). The stereotactic implantation of the electrodes (3391, 3387 or B3301533; Medtronic plc, Dublin, Ireland) and the implantation of the internal pulse generator (IPG) (ActivaRC or PerceptPC; Medtronic plc, Dublin, Ireland) was performed in one setting with the patient under general anesthesia. Postoperatively, the position of the electrode was verified using CT scans with a slice thickness of 1mm, which were integrated with MR imaging.

### Stimulation

Stimulation typically started 6-8 weeks post-surgery and was adjusted by a psychiatrist experienced in DBS. Bilateral stimulation of each of the four contacts was initially assessed for tolerability and efficacy. Subsequently, the optimal contact voltage was gradually increased to attain maximum therapeutic efficacy. Upon achieving optimal voltage, subsequent optimization of additional stimulation parameters, including frequency and pulse width, was conducted. If the target effectiveness was not achieved, the same procedure was conducted with the second-best contact.

### Assessment tools

Y-BOCS was used to evaluate the existence and severity of OCD symptoms (55), which measures the severity of symptoms of OCD based on scores of obsessions and compulsions. The Y-BOCS comprises ten items that assess the severity and influence of both obsessions and compulsions. Each of the ten items is evaluated on a five-point scale ranging from zero to four, as follows: No symptoms are indicated by a value of 0, while extreme symptoms are defined by a value of 4. The Y-BOCS has a maximal possible score of 40. It is divided into the following categories: Obsessions subscale (Items 1–5): Scores range from 0 to 20. Compulsions subscale (Items 6–10): Scores range from 0 to 20.

The scores are generally interpreted with the following criteria: 0–7: Subclinical or no symptoms, 8–15: Mild symptoms of OCD, 16–23: Moderate symptoms of OCD, 24–31: Symptoms of OCD that are severe, 32–40: Severe symptoms of OCD.

For IBS-symptoms, we used the Gastrointestinal Symptom Rating Scale for Irritable Bowel Syndrome (GSRS-IBS) (56), in its German version (Reizdarm-Fragebogen RDF), which proves to be an effective, reliable, and valid questionnaire for the assessment of symptom severity in IBS (57). The GSRS-IBS is a 13-item measure of gastrointestinal symptom severity for the last week. The items measure severity of abdominal pain (Item 1), pain relieved by a bowel action (Item 2), bloating (Item 3), passing gas (Item 4), constipation (Item 5), diarrhea (Item 6), loose stools (Item 7), hard stools (Item 8), urgent need for bowel movement (Item 9), incomplete bowel emptying (Item 10), fullness shortly after meal (Item 11), fullness long after eating (Item 12), and visible distension (Item 13). The items are scored between 1 and 7, where 1 corresponds to “no discomfort at all” and 7 to “very severe discomfort” from the symptom (58).

The GSRS-IBS is not designed to serve as a diagnostic instrument for IBS, but rather for evaluating the severity of symptoms over time. In general, a higher total score indicates more severe symptoms. In our analysis, we considered scores of 32 and above as an indicator of clinically relevant symptom severity, as this cut-off score has been shown good levels of sensitivity and acceptable levels of specificity (59).

### Analysis of results

All statistical analyses were performed using R (version 4.3.2) and R-Studio (2023.12.1 Build 402) with the nlme package (version 3.1-166 (60) Pinheiro et al., 2021).

To evaluate symptom changes in Y-BOCS and GSRS-IBS scores over study visits (baseline, before stimulation, optimized stimulation, 3 months follow-up, 6 months follow-up, 9 months follow-up, and 12 months follow-up), linear mixed effects models were applied. Models were estimated using restricted maximum likelihood estimation (REML) without specific imputation of missing values, assuming data were missing at random (MAR). In these analyses, study visit was treated as a fixed effect and the individual patient as a random effect. In case of a significant effect of study visit (tested using the expected mean squares approach), *post-hoc* pairwise comparisons of the fixed effects were performed. In case of the Y-BOCS scale, *post hoc* results were adjusted using the Tukey method. For the GSRS-IBS, *post hoc* testing of differences between time points was conducted irrespective of overall significance for the fixed effect and without adjustment of p-values for multiple comparisons, meaning each comparison was independently tested for significance without correction for cumulative error probability. The level for statistical significance was set at 5%. The liberal testing of significant differences between time points for the GSRS-IBS was chosen due to the exploratory nature of the study and the restricted sample size. The primary goal was to identify potential trends and relationships that warrant further investigation in future, more targeted studies. Adjusting the alpha level might increase the risk of Type II errors, potentially obscuring meaningful findings in this early phase of research. As such, we present the results without correction to avoid an overly conservative approach, but acknowledge that any significant findings should be interpreted with caution and confirmed in subsequent confirmatory analyses. Additionally, we performed a bivariate correlation analysis using Spearman’s rho in order to investigate the associations between the Y-BOCS scale scores and the GSRS-IBS for each of the study visits.

## Results

Since not all of our patients were investigated with GSRS-IBS, we are reporting in this study only the results of those 9 patients who completed the questionnaire (4 males, 5 females; age between 24 and 61 years, mean age: 39.1 ± 11.5 years).

The results are primarily presented in a descriptive manner due to the small sample size. For detailed scores over different timepoints, please see **Supplementary Materials**.

### GSRS-IBS

#### Descriptive analysis

Only three patients scored more than 32 points at baseline. As shown in **Table 1**, the mean score did not differ substantially between baseline assessment (27.78) and the optimization of stimulation (24.71), however there was a descriptive trend toward score reduction throughout the study period and until the last follow up visit after 12 months (23.88). As can also be seen from the Table, there were slight fluctuations in the number of completed questionnaires across the study period (see **Table 1**).

**Table 1:** GSRS-IBS mean scores over study visits.

Timepoint	All patients			Patients with IBS		
	Mean	SD	n	Mean	SD	n
GSRS-IBS_BL	27.78	12.25	9	42.33	8.62	3
GSRS-IBS_BS	25.88	12.92	8	41.50	10.61	2
GSRS-IBS_OS	24.71	7.16	7	30.50	4.95	2
GSRS-IBS_3m	25.11	13.20	9	36.33	13.65	3
GSRS-IBS_6m	26.00	12.11	9	38.67	6.66	3
GSRS-IBS_9m	22.50	9.10	8	30.33	7.51	3
GSRS-IBS_12m	23.88	10.56	8	31	11.36	3

BL, Baseline; BS, Before stimulation; OS, Optimized stimulation; 3-12m, Follow-up visit 3/6/9/12 months after optimization of stimulation.

Three patients were scoring 32 points or more at baseline, and all of them showed a reduction of the GSRS-IBS score at the last follow-up (12% (patient 1), 30% (patient 7), and 40% (patient 6)). Among the other 6 patients, five showed decreased scores at last follow-up.

**Figure 1:**
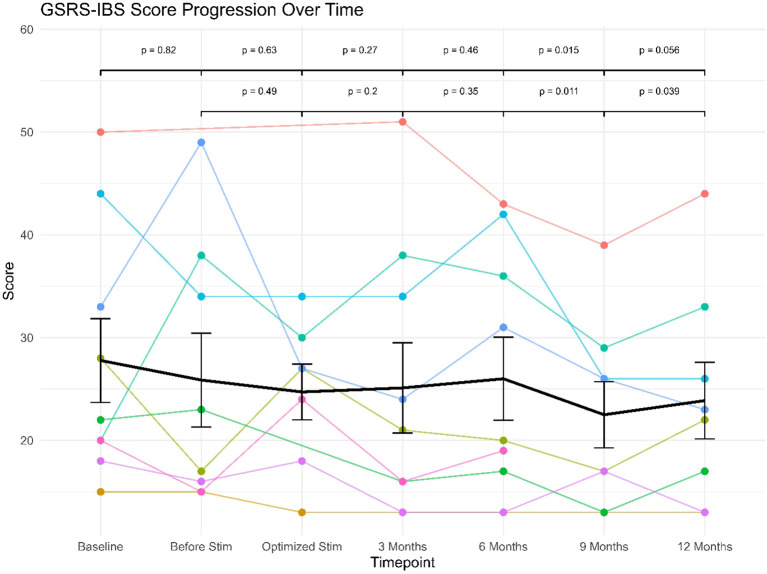
GSRS-IBS scores progression over follow-up for individual patients (star symbols mark patients displaying clinical levels of IBS).

The linear mixed effects model revealed no significant (fixed) effect of study visit (F(6, 43) = 1.90), p >.10). The (fixed) effect of study visit (marginal R^2^= .037) explained only 3.7% of variance, while the specified model explained a total of 82.4% of variance in the data (conditional R^2^ = .824), implying strong effects of the (random) effect of patient (i.e. large interindividual differences in the scale scores). However, exploratory *post-hoc* comparisons between study visits showed significant or near-significant differences between GSRS-IBS scores between study visits at baseline or before stimulation versus those conducted at the 9 or 12 month follow-ups (see **Figure 1**). Re-running the model in the subsample of three patients with clinical GSRS-IBS scores (≥ 32) at baseline yielded similar results: the fixed effect of study visit remained non-significant (F(6, 10) = 2.25, p >.12). However, in this subsample, marginal R^2^ increased to.214, indicating that study visit accounted for 21.4% of variance—a notable increase compared to the full sample. The conditional R^2^ = .719 showed that the full model still explained 71.9% of variance, reinforcing the strong interindividual differences in scale scores driven by the random effect of patient.

### Y-BOCS

#### Descriptive analysis

The 9 patients had baseline scores ranging from 23 to 37 points. Regarding severity according to Y-BOCS, one patient of them was showing moderate symptoms, three patients were showing severe symptoms, while five patients were showing extreme symptoms. After optimizing the stimulation, the mean Y-BOCS score decreased from 31.11 at baseline to 16.89 after the optimization of stimulation. Furthermore, the reduction in Y-BOCS score remained stable through following visits to reach 16.50 at the last follow-up.

The treatment response was defined as a reduction in YBOCS of at least 35% compared to baseline, in accordance with established standards (61). Out of the 9 patients, 7 of them (78%) were responders with Y-BOCS score decreases ranging from 37% to 74%. All three patients displaying clinical levels of IBS were responders with Y-BOCS score decreases ranging from 45% to 62% (see **Table 2**).

**Table 2:** Y-BOCS mean scores over study visits.

Timepoint	All patients			Patients with IBS		
	Mean	SD	n	Mean	SD	n
YBOCS_BL	31.11	4.78	9	32	5,57	3
YBOCS_BS	31.44	3.05	9	32,33	3,06	3
YBOCS_OS	16.89	6.79	9	16	7	3
YBOCS_3m	16.78	7.43	9	14,33	2,89	3
YBOCS_6m	16.00	7.57	9	15,33	0,58	3
YBOCS_9m	16.13	8.87	8	14,67	1,53	3
YBOCS_12m	16.50	8.45	8	15,33	2,31	3

BL, Baseline; BS, Before stimulation; OS, Optimized stimulation; 3-12m, Follow-up visit 3/6/9/12 months after optimization of stimulation.

**Figure 2:**
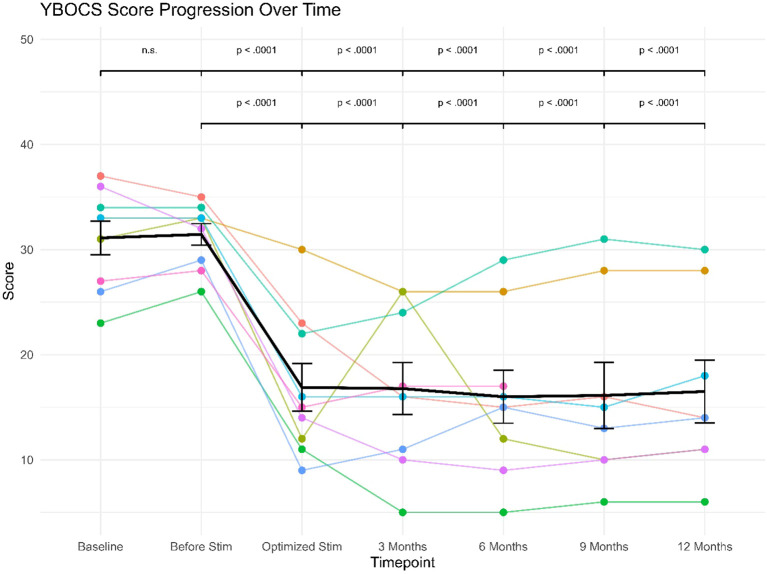
Y-BOCS’ scores progression over follow-up for individual patients (star symbols mark patients displaying clinical levels of IBS).

The linear mixed effects model revealed a significant (fixed) effect of study visit (F(6, 43) = 27.54), p <.0001). The (fixed) effect of study visit explained 50.1% of variance (marginal R^2^= .501), while the specified model explained a total of 81.9% of variance in the data (conditional R^2^ = .819). The conducted *post-hoc* comparisons between study visits showed highly significant differences in Y-BOCS scores between study visits at baseline or before stimulation versus all subsequent study visits (see **Figure 2**), since Y-BOCS scores dropped considerably with optimization of the deep brain stimulation. As can also be seen from the Figure, this drop in Y-BOCS scores was stable at the level of the individual patients.

### Correlation between GSRS-IBS and Y-BOCS

In order to test for potential associations between OCD and IBS symptoms, bivariate correlations between the GSRS-IBS and Y-BOCS scale scores were calculated for each of the study visit timepoints (see **Table 3**). As can be seen, there were negligible associations between scale scores both before the beginning of DBS treatment as well as throughout the first half of the follow-up period (i.e. until the 6-month follow-up visit). Following this, however, there was an increase in the strength of association and moderate-to-large correlations between scale scores could be observed at both the 9-month and the 12-month follow-up visit (.38 -.51, see **Table 3**). However, none of these associations was statistically significant (all p’s >.19).

**Table 3:** Correlations between GSRS-IBS and YBOCS over study visits.

Timepoint	*r_S_*	p-value	n
**BL**	-.01	.98	9
**BS**	.17	.68	8
**OS**	-.09	.85	7
**3m**	.06	.87	9
**6m**	.24	.53	9
**9m**	.51	.19	8
**12m**	.38	.35	8

r_S_, Spearman’s rho; BL, Baseline; BS, Before stimulation; OS, Optimized stimulation; 3-12m, Follow-up visit 3/6/9/12 months after optimization of stimulation.

## Discussion

Irritable bowel syndrome (IBS) is a chronic functional gastrointestinal disorder that affects 9%-23% of the population across the world. It can be both physically and emotionally debilitating, making it difficult for people to work, socialize, and even take care of daily activities (62).

While drug therapy has successfully induced remission in many cases of IBS, its effectiveness is limited and it may result in significant adverse effects (63).

Given the rising prevalence of psychological disorders in gastroenterology, innovative strategies are necessary to enhance the management of patients with IBS (27). Both patients and clinicians have actively sought alternate therapeutic options, which has led to an increasing interest in neuromodulative approaches within the IBS-research field (64, 65). For example, some studies have been conducted to assess the potential of vagus nerve stimulation (VNS) in this area depending on its anti-inflammatory effects, in both its invasive (66–69), or non-invasive variant (70, 71), yielding promising findings. Also, other neurostimulative methods like invasive sacral nerve stimulation (72, 73), non-invasive tibial nerve stimulation (74), auricular neurostimulation (75), transcutaneous electrical acustimulation (76), and transcranial magnetic stimulation (77) have been suggested.

Both OCD and IBS are diseases which often coexist together, and could also be exacerbated by increased stress and anxiety. The explanation of the pathogenesis of IBS has been often linked with chronic stress as a role factor (78, 79). The link between psychological disturbances and the digestive tract in the so called brain–gut axis appear to be primarily modulated by the autonomic nervous system (79, 80). Acute or chronic stress, even in healthy persons, causes the autonomic nervous system to release corticotrophin-releasing factor, which is known to disrupt gut function and may consequently result in gastrointestinal symptoms (81). In IBS, the HPA axis becomes dysregulated (79), leading the individual to be more susceptible to, and less able to recover from, stressful events (82). This dysregulation HPA axis is also known to have a role in many psychiatric disorders like OCD and depression (83, 84), which could explain the frequent co-existence of such disorders with IBS (27). Studies have shown that IBS patients have altered activity in brain regions associated with pain processing and emotion regulation (85, 86). It has been suggested that the parts of the brain that regulate visceral pain are located in the central amygdala, hippocampus, BNST and locus coeruleus, while the brain regions located in hypothalamus, amygdala, and dorsal raphe nucleus (DRN) affect psychological state (87).

For OCD, stress is also believed to play an important role. Research indicated that stress may serve as both a triggering and aggravating factor for OCD-symptoms (88), demonstrating that stress has obvious effects on brain regions or circuits that are involved in the pathogenesis of OCD, like corticostriatal and limbic circuitry. For instance, stress can lead to neuronal atrophy in frontal cortices, the dorsomedial striatum, and the hippocampus as well as neuronal hypertrophy in the dorsolateral striatum putamen and amygdala (89).

As mentioned before, deep brain stimulation for OCD has been studied in treatment-resistant patients targeting different brain regions. For DBS in OCD, the BNST is considered to be a reliable implanting target option, yielding satisfying results. Many studies have shown that BNST-DBS for OCD can be effective (54, 90–92), comparable to other brain targets (93) or even with better outcome (43, 94).

The BNST is considered as a part of the “extended amygdala” (95), and believed to be involved in striatal circuitry that integrates descending glutamatergic input with ascending modulatory inputs (96). The BNST plays a crucial role in linking limbic forebrain structures to hypothalamus and brainstem regions involved in autonomic and neuroendocrine functions, hence facilitating the integration of physiological and behavioral responses (97).

In this study we investigated treatment resistant patients with severe OCD, who underwent DBS treatment with respect to IBS symptoms.

In this group three patients (33%) had a GSRS-IBS score of at least 32 corresponding to a significant impairment by co-morbid IBS symptoms, which is in the expected range of IBS comorbidity among OCD patients (30–32). The other patients had GSRS-IBS baseline scores between 15 and 28. In the majority of patients IBS symptoms fluctuated over the course of treatment with a slight tendency toward improvement. In all but one patient the GSRS-IBS score at the end of follow-up was lower than at baseline. The patient whose IBS symptoms worsened did not respond to DBS regarding his OCD symptoms. Importantly, OCD symptoms decreased in all treated patients, with symptom decreases of at least 35% of Y-BOCS scores occurring in 7 out of 9 patients. We found moderate-to-large correlations between scores in the Y-BOCS and the GSRS-IBS at the 9 month and 12 month follow-up visits (see **Table 3**), albeit these associations did not reach statistical significance which may be due to the small sample size.

The lack of statistical significance has to be put into perspective of the small sample size in which only 3 patients exhibited clinically relevant IBS symptoms. The small sample size, particularly with only three patients meeting criteria for clinically relevant IBS symptoms, naturally limits the generalizability and robustness of our conclusions, necessitating cautious interpretation. Thus, with our data we can neither confirm nor exclude an effect of DBS on IBS symptoms. Whereas IBS symptoms showed relatively large fluctuations over time in most participants and were only very loosely correlated with OCD symptoms at the beginning, the correlation between the GSRS-IBS and the Y-BOCS scores tended to increase and were largest at the 9 months and 12 months follow-up visits. One may speculate that the tendency toward IBS symptom reduction and toward increased correlation between OCD and IBS symptoms over the course of treatment might reflect an effect of DBS on IBS symptoms. Whether this may be a direct effect of stimulation or whether the effects are mediated via OCD symptom improvement remains speculative as well. Furthermore, it is important to acknowledge the possibility that gastrointestinal symptom changes observed in some patients may not solely reflect therapeutic benefits but could also represent complications or side effects of the DBS treatment process. This perspective highlights the complexity of interpreting symptom changes and reinforces the need for future studies to carefully distinguish between potential therapeutic effects and side effects.

Notably, the observation of gastrointestinal side effects further underscores, that stimulation of the NAc-ALIC region can have an impact on gastrointestinal regulation. Whether stimulation results in beneficial effects or in adverse effects may depend on the individual’s symptomatology and related brain activity. One could imagine that IBS related increased connectivity can be disrupted by DBS, which then results in symptom reduction, whereas disruption of physiological brain activity in patients without IBS might cause gastrointestinal side effects.

Since the reported correlations were statistically insignificant (despite medium-to-large effect sizes, see **Table 3**), they should be considered as preliminary and clearly need to be replicated and extended in future, larger trials. Given the limited statistical power inherent in such a small cohort, even medium-to-large effect sizes may not have reached statistical significance. Therefore, these results should be regarded as exploratory and hypothesis-generating rather than conclusive.

Nevertheless, our findings support the notion that DBS exhibits an effect on comorbid IBS symptoms in OCD patients and warrants further investigations of this topic. It also has to be considered that the stimulation protocol was optimized in every individual patient in order to achieve maximal reduction of OCD symptoms (see **Table 4**). We cannot exclude that other stimulation parameters and especially other contacts at the stimulation electrode might have been more successful for reduction of IBS symptoms, as suggested in a previous case report (53). This raises the broader question of whether the BNST represents the “optimal” target for patients with co-occurring OCD and IBS symptoms. To investigate these possibilities more robustly, future studies with larger sample sizes are needed, ideally encompassing more patients with more severe IBS symptoms. Future studies could benefit from multi-center collaborations to recruit a larger, more representative cohort or by specifically targeting patients with both severe OCD and IBS symptoms to better assess the effects of DBS.

**Table 4:** Individual stimulation’s parameter for each patient.

Patient	Sex	Year of Surgery	DBS parameters (at LFU*)	Active Contacts
			Amplitude, Frequency, pulse width	
1	♂	2021	5,8V, 160Hz, 60ms	0 & 8
2	♂	2021	5,5V, 130Hz, 120 ms	0 & 8
3	♀	2021	4,6V, 160Hz, 120ms	1 & 9
4	♀	2022	4V, 170Hz, 80ms	0 & 8
5	♂	2022	4,7V, 130Hz, 120ms	0 & 8
6	♀	2022	4,8V, 150Hz, 70ms	0 & 8
7	♀	2022	4,6V, 130Hz, 60ms	3 & 11
8	♀	2023	4,5V, 130Hz, 60ms	1 & 9
9	♂	2023	5,5V, 130Hz, 60ms	2 & 10

*LFU, last follow-up.

## Data Availability

The original contributions presented in the study are included in the article/**Supplementary Material**. Further inquiries can be directed to the corresponding author.

## References

[1] Canavan C West J Card T. The epidemiology of irritable bowel syndrome. Clin Epidemiol. (2014) 6:71–80. doi: 10.2147/CLEP.S40245 24523597 PMC3921083

[2] Ringel Y Sperber AD Drossman DA. Irritable bowel syndrome. Annu Rev Med. (2001) 52:319–38. doi: 10.1146/annurev.med.52.1.319 11160782

[3] Drossman DA Camilleri M Mayer EA Whitehead WE. AGA technical review on irritable bowel syndrome. Gastroenterology. (2002) 123:2108–31. doi: 10.1053/gast.2002.37095 12454866

[4] Longstreth GF Wilson A Knight K Wong J Chiou CF Barghout V. Irritable bowel syndrome, health care use, and costs: a U.S. managed care perspective. Am J Gastroenterol. (2003) 98:600–7. doi: 10.1111/j.1572-0241.2003.07296.x 12650794

[5] Occhipinti K Smith JW. Irritable bowel syndrome: a review and update. Clin Colon Rectal Surg. (2012) 25:46–52. doi: 10.1055/s-0032-1301759 23449495 PMC3348735

[6] Horwitz BJ Fisher RS. The irritable bowel syndrome. N Engl J Med. (2001) 344:1846–50. doi: 10.1056/NEJM200106143442407 11407347

[7] Halmos EP Power VA Shepherd SJ Gibson PR Muir JG. A diet low in FODMAPs reduces symptoms of irritable bowel syndrome. Gastroenterology. (2014) 146:67–75.e5. doi: 10.1053/j.gastro.2013.09.046 24076059

[8] Chang JY Talley NJ. An update on irritable bowel syndrome: from diagnosis to emerging therapies. Curr Opin Gastroenterol. (2011) 27:72–8. doi: 10.1097/MOG.0b013e3283414065 21099429

[9] Mayer EA Naliboff BD Chang L. Basic pathophysiologic mechanisms in irritable bowel syndrome. Dig Dis. (2001) 19:212–8. doi: 10.1159/000050682 11752839

[10] Soares RL. Irritable bowel syndrome: a clinical review. World J Gastroenterol. (2014) 20:12144–60. doi: 10.3748/wjg.v20.i34.12144 PMC4161800 25232249

[11] Tanaka Y Kanazawa M Fukudo S Drossman DA. Biopsychosocial model of irritable bowel syndrome. J Neurogastroenterol Motil. (2011) 17:131–9. doi: 10.5056/jnm.2011.17.2.131 PMC3093004 21602989

[12] Drossman DA Li Z Andruzzi E Temple RD Talley NJ Thompson WG. U.S. householder survey of functional gastrointestinal disorders. Prevalence, sociodemography, and health impact. Dig Dis Sci. (1993) 38:1569–80. doi: 10.1007/BF01303162 8359066

[13] Mertz H Morgan V Tian H Nicolson M Kufner K Schwartz S. Altered rectal perception is a biological marker of patients with irritable bowel syndrome. Gastroenterology. (1995) 109:40–52. doi: 10.1016/0016-5085(95)90267-8 7797041

[14] Keszthelyi D Troost FJ Simrén M Ludidi S Kruimel JW Conchillo. Revisiting concepts of visceral nociception in irritable bowel syndrome. Eur J Pain. (2012) 16:1444–54. doi: 10.1002/j.1532-2149.2012.00147.x 22504901

[15] Azpiroz F Bouin M Camilleri M Mayer EA Poitras P Serra J. Mechanisms of hypersensitivity in IBS and functional disorders. Neurogastroenterol Motil. (2007) 19:62–88. doi: 10.1111/j.1365-2982.2006.00875.x 17280586

[16] Coss-Adame E Rao SS. Brain and gut interactions in irritable bowel syndrome: new paradigms and new understandings. Curr Gastroenterol Rep. (2014) 16:379. doi: 10.1007/s11894-014-0379-z 24595616 PMC4083372

[17] Aziz Q Thompson DG. Brain-gut axis in health and disease. Gastroenterology. (1998) 114:559–78. doi: 10.1016/S0016-5085(98)70540-2 9496948

[18] Gaman A Kuo B. Neuromodulatory processes of the brain-gut axis. Neuromodulation. (2008) 11:249–59. doi: 10.1111/j.1525-1403.2008.00172.x PMC2763396 19844605

[19] Weaver KR Sherwin LB Walitt B Melkus GD Henderson WA. Neuroimaging the brain-gut axis in patients with irritable bowel syndrome. World J Gastrointest Pharmacol Ther. (2016) 7:320–33. doi: 10.4292/wjgpt.v7.i2.320 PMC4848255 27158548

[20] Berman SM Naliboff BD Suyenobu B Labus JS Stains J Ohning G. Reduced brainstem inhibition during anticipated pelvic visceral pain correlates with enhanced brain response to the visceral stimulus in women with irritable bowel syndrome. J Neurosci. (2008) 28:349–59. doi: 10.1523/JNEUROSCI.2500-07.2008 PMC6670525 18184777

[21] Price JL. Comparative aspects of amygdala connectivity. Ann N Y Acad Sci. (2003) 985:50–8. doi: 10.1111/j.1749-6632.2003.tb07070.x 12724147

[22] Wilder-Smith CH. The balancing act: endogenous modulation of pain in functional gastrointestinal disorders. Gut. (2011) 60:1589–99. doi: 10.1136/gutjnl-2011-300253 21768212

[23] Tillisch K Mayer EA Labus JS. Quantitative meta-analysis identifies brain regions activated during rectal distension in irritable bowel syndrome. Gastroenterology. (2011) 140:91–100. doi: 10.1053/j.gastro.2010.07.053 20696168 PMC3253553

[24] Pae CU Masand PS Ajwani N Lee C Patkar AA. Irritable bowel syndrome in psychiatric perspectives: a comprehensive review. Int J Clin Pract. (2007) 61:1708–18. doi: 10.1111/j.1742-1241.2007.01409.x 17877658

[25] Zamani M Alizadeh-Tabari S Zamani V. Systematic review with meta-analysis: the prevalence of anxiety and depression in patients with irritable bowel syndrome. Aliment Pharmacol Ther. (2019) 50:132–43. doi: 10.1111/apt.2019.50.issue-2 31157418

[26] Banerjee A Sarkhel S Dhali GK Paul I Das A. A follow-up study of anxiety and depressive symptoms in irritable bowel syndrome. Indian J Psychiatry. (2024) 66:142–7. doi: 10.4103/indianjpsychiatry.indianjpsychiatry_732_23 PMC10956588 38523769

[27] Staudacher HM Black CJ Teasdale SB Mikocka-Walus A Keefer L. Irritable bowel syndrome and mental health comorbidity - approach to multidisciplinary management. Nat Rev Gastroenterol Hepatol. (2023) 20:582–96. doi: 10.1038/s41575-023-00794-z PMC10237074 37268741

[28] Aguglia A Signorelli MS Albert U Maina G. The impact of general medical conditions in obsessive-compulsive disorder. Psychiatry Investig. (2018) 15:246–53. doi: 10.30773/pi.2017.06.17.2 PMC5900370 29475243

[29] Fakhraei B Firouzabadi A Farjam M Fattahi M Kazemi M Naini M. Frequency of different psychiatric disorders in patients with functional bowel disorders: A short report. Ann Colorectal Res. (2015) 3. doi: 10.17795/acr-27621

[30] Davarinejad O RostamiParsa F Radmehr F Farnia V Alikhani M. The prevalence of obsessive-compulsive disorder in patients with irritable bowel syndrome: A cross-sectional study. J Educ Health Promot. (2021) 10:50. doi: 10.4103/jehp.jehp_812_20 34084797 PMC8057190

[31] Gros DF Antony MM McCabe RE Swinson RP. Frequency and severity of the symptoms of irritable bowel syndrome across the anxiety disorders and depression. J Anxiety Disord. (2009) 23:290–6. doi: 10.1016/j.janxdis.2008.08.004 18819774

[32] Masand PS Keuthen NJ Gupta S Virk S Yu-Siao B Kaplan D. Prevalence of irritable bowel syndrome in obsessive-compulsive disorder. CNS Spectr. (2006) 11:21–5. doi: 10.1017/S1092852900024123 16400252

[33] North CS Napier M Alpers DH Spitznagel EL. Complaints of constipation in obsessive-compulsive disorder. Ann Clin Psychiatry. (1995) 7:65–70. doi: 10.3109/10401239509149029 8556095

[34] Turna J Grosman Kaplan K Patterson B Bercik P Anglin R Soreni N. Higher prevalence of irritable bowel syndrome and greater gastrointestinal symptoms in obsessive-compulsive disorder. J Psychiatr Res. (2019) 118:1–6. doi: 10.1016/j.jpsychires.2019.08.004 31437616

[35] Denys D. Pharmacotherapy of obsessive-compulsive disorder and obsessive-compulsive spectrum disorders. Psychiatr Clin North Am. (2006) 29:553–84, xi. doi: 10.1016/j.psc.2006.02.013 16650723

[36] Eddy KT Dutra L Bradley R Westen D. A multidimensional meta-analysis of psychotherapy and pharmacotherapy for obsessive-compulsive disorder. Clin Psychol Rev. (2004) 24:1011–30. doi: 10.1016/j.cpr.2004.08.004 15533282

[37] Fineberg NA Brown A Reghunandanan S Pampaloni I. Evidence-based pharmacotherapy of obsessive-compulsive disorder. Int J Neuropsychopharmacol. (2012) 15:1173–91. doi: 10.1017/S1461145711001829 22226028

[38] Perlmutter JS Mink JW. Deep brain stimulation. Annu Rev Neurosci. (2006) 29:229–57. doi: 10.1146/annurev.neuro.29.051605.112824 PMC4518728 16776585

[39] H.d.e. Available from: humanitarian device exemption (hde). fda.gov (2009). Available online at: https://www.frontiersin.org/journals/psychiatry/articles/10.3389/fpsyt.2023.1242566/full.

[40] Abelson JL Curtis GC Sagher O Albucher RC Harrigan M Taylor SF. Deep brain stimulation for refractory obsessive-compulsive disorder. Biol Psychiatry. (2005) 57:510–6. doi: 10.1016/j.biopsych.2004.11.042 15737666

[41] Menchón JM Real E Alonso P Aparicio MA Segalas C Plans G. A prospective international multi-center study on safety and efficacy of deep brain stimulation for resistant obsessive-compulsive disorder. Mol Psychiatry. (2021) 26:1234–47. doi: 10.1038/s41380-019-0562-6 PMC7985042 31664175

[42] Nuttin B Cosyns P Demeulemeester H Gybels J Meyerson B. Electrical stimulation in anterior limbs of internal capsules in patients with obsessive-compulsive disorder. Lancet. (1999) 354:1526. doi: 10.1016/S0140-6736(99)02376-4 10551504

[43] Luyten L Hendrickx S Raymaekers S Gabriëls L Nuttin B. Electrical stimulation in the bed nucleus of the stria terminalis alleviates severe obsessive-compulsive disorder. Mol Psychiatry. (2016) 21:1272–80. doi: 10.1038/mp.2015.124 26303665

[44] Goodman WK Foote KD Greenberg BD Ricciuti N Bauer R Ward H. Deep brain stimulation for intractable obsessive compulsive disorder: pilot study using a blinded, staggered-onset design. Biol Psychiatry. (2010) 67:535–42. doi: 10.1016/j.biopsych.2009.11.028 PMC5796545 20116047

[45] Greenberg BD Gabriels LA Malone DA Jr Rezai AR Friehs GM Okun MS. Deep brain stimulation of the ventral internal capsule/ventral striatum for obsessive-compulsive disorder: worldwide experience. Mol Psychiatry. (2010) 15:64–79. doi: 10.1038/mp.2008.55 18490925 PMC3790898

[46] Tyagi H Apergis-Schoute AM Akram H Foltynie T Limousin P Drummond LM. A randomized trial directly comparing ventral capsule and anteromedial subthalamic nucleus stimulation in obsessive-compulsive disorder: clinical and imaging evidence for dissociable effects. Biol Psychiatry. (2019) 85:726–34. doi: 10.1016/j.biopsych.2019.01.017 PMC6467837 30853111

[47] Barcia JA Avecillas-Chasín JM Nombela C Arza R García-Albea J Pineda-Pardo JA. Personalized striatal targets for deep brain stimulation in obsessive-compulsive disorder. Brain Stimul. (2019) 12:724–34. doi: 10.1016/j.brs.2018.12.226 30670359

[48] Denys D Mantione M Figee M van den Munckhof P Koerselman F Westenberg H. Deep brain stimulation of the nucleus accumbens for treatment-refractory obsessive-compulsive disorder. Arch Gen Psychiatry. (2010) 67:1061–8. doi: 10.1001/archgenpsychiatry.2010.122 20921122

[49] Huff W Lenartz D Schormann M Lee SH Kuhn J Koulousakis A. Unilateral deep brain stimulation of the nucleus accumbens in patients with treatment-resistant obsessive-compulsive disorder: Outcomes after one year. Clin Neurol Neurosurg. (2010) 112:137–43. doi: 10.1016/j.clineuro.2009.11.006 20006424

[50] Mallet L Polosan M Jaafari N Baup N Welter ML Fontaine D. Subthalamic nucleus stimulation in severe obsessive-compulsive disorder. N Engl J Med. (2008) 359:2121–34. doi: 10.1056/NEJMoa0708514 19005196

[51] Cruz S Gutiérrez-Rojas L González-Domenech P Díaz-Atienza F Martínez-Ortega JM Jiménez-Fernández S. Deep brain stimulation in obsessive-compulsive disorder: Results from meta-analysis. Psychiatry Res. (2022) 317:114869. doi: 10.1016/j.psychres.2022.114869 36240634

[52] Hageman SB van Rooijen G Bergfeld IO Schirmbeck F de Koning P Schuurman PR. Deep brain stimulation versus ablative surgery for treatment-refractory obsessive-compulsive disorder: A meta-analysis. Acta Psychiatr Scand. (2021) 143:307–18. doi: 10.1111/acps.v143.4 33492682

[53] Langguth B Sturm K Wetter TC Lange M Gabriels L Mayer EA. Deep brain stimulation for obsessive compulsive disorder reduces symptoms of irritable bowel syndrome in a single patient. Clin Gastroenterol Hepatol. (2015) 13:1371–1374.e3. doi: 10.1016/j.cgh.2015.01.023 25638586 PMC4986991

[54] Abdelnaim MA Lang-Hambauer V Hebel T Schoisswohl S Schecklmann M Deuter D. Deep brain stimulation for treatment resistant obsessive compulsive disorder; an observational study with ten patients under real-life conditions. Front Psychiatry. (2023) 14:1242566. doi: 10.3389/fpsyt.2023.1242566 37779611 PMC10533930

[55] Goodman WK Price LH Rasmussen SA Mazure C Fleischmann RL Hill CL. The Yale-Brown Obsessive Compulsive Scale. I. Development, use, and reliability. Arch Gen Psychiatry. (1989) 46:1006–11. doi: 10.1001/archpsyc.1989.01810110048007 2684084

[56] Wiklund IK Fullerton S Hawkey CJ Jones RH Longstreth GF Mayer EA. An irritable bowel syndrome-specific symptom questionnaire: development and validation. Scand J Gastroenterol. (2003) 38:947–54. doi: 10.1080/00365520310004209 14531531

[57] Schäfer SK Weidner KJ Hoppner J Becker N Friedrich D Stokes CS. Design and validation of a German version of the GSRS-IBS - an analysis of its psychometric quality and factorial structure. BMC Gastroenterol. (2017) 17:139. doi: 10.1186/s12876-017-0684-8 29202711 PMC5715554

[58] Ljótsson B Jones M Talley NJ Kjellström L Agréus L Andreasson A. Discriminant and convergent validity of the GSRS-IBS symptom severity measure for irritable bowel syndrome: A population study. United Eur Gastroenterol J. (2020) 8:284–92. doi: 10.1177/2050640619900577 PMC7184654 32213021

[59] Schäfer SK Weidner KJ Becker N Lass-Hennemann J Stokes C Lammert F. Sensitivity and specificity of the reizdarm-fragebogen. Psychother Psychosom Med Psychol. (2019) 69:382–8. doi: 10.1055/a-0834-6207 30731510

[60] Pinheiro J Bates D DebRoy S Sarkar D R Core Team. nlme: Linear and Nonlinear Mixed Effects Models (2021). Available online at: https://CRAN.R-project.org/package=nlme (Accessed November 05, 2024).

[61] Farris SG McLean CP Van Meter PE Simpson HB Foa EB. Treatment response, symptom remission, and wellness in obsessive-compulsive disorder. J Clin Psychiatry. (2013) 74:685–90. doi: 10.4088/JCP.12m07789 PMC3959901 23945445

[62] Saha L. Irritable bowel syndrome: pathogenesis, diagnosis, treatment, and evidence-based medicine. World J Gastroenterol. (2014) 20:6759–73. doi: 10.3748/wjg.v20.i22.6759 PMC4051916 24944467

[63] Cheng J Shen H Chowdhury R Abdi T Selaru F Chen JDZ. Potential of electrical neuromodulation for inflammatory bowel disease. Inflammation Bowel Dis. (2020) 26:1119–30. doi: 10.1093/ibd/izz289 31782957

[64] Abell TL Chen J Emmanuel A Jolley C Sarela AI Törnblom H. Neurostimulation of the gastrointestinal tract: review of recent developments. Neuromodulation. (2015) 18:221–7. doi: 10.1111/ner.12260 PMC4383702 25581846

[65] Alam MJ Chen JDZ. Non-invasive neuromodulation: an emerging intervention for visceral pain in gastrointestinal disorders. Bioelectron Med. (2023) 9:27. doi: 10.1186/s42234-023-00130-5 37990288 PMC10664460

[66] Clarençon D Pellissier S Sinniger V Kibleur A Hoffman D Vercueil L. Long term effects of low frequency (10 hz) vagus nerve stimulation on EEG and heart rate variability in Crohn's disease: a case report. Brain Stimul. (2014) 7:914–6. doi: 10.1016/j.brs.2014.08.001 25263316

[67] Bonaz B Sinniger V Hoffmann D Clarençon D Mathieu N Dantzer C. Chronic vagus nerve stimulation in Crohn's disease: a 6-month follow-up pilot study. Neurogastroenterol Motil. (2016) 28:948–53. doi: 10.1111/nmo.2016.28.issue-6 26920654

[68] D’Haens G Cabrijan Z Eberhardson M Berg R Löwenberg M Danese S. 367 – vagus nerve stimulation reduces disease activity and modulates serum and autonomic biomarkers in biologicrefractory crohn's disease. Gastroenterology. (2019) 156:S–75. doi: 10.1016/S0016-5085(19)36973-2

[69] Kibleur A Pellissier S Sinniger V Robert J Gronlier E Clarençon D. Electroencephalographic correlates of low-frequency vagus nerve stimulation therapy for Crohn's disease. Clin Neurophysiol. (2018) 129:1041–6. doi: 10.1016/j.clinph.2018.02.127 29573733

[70] Mion F Pellissier S Garros A. Transcutaneous auricular vagus nerve stimulation for the treatment of irritable bowel syndrome: a pilot, open-label study. Bioelectronics Med. (2020) 3:5–12. doi: 10.2217/bem-2020-0004

[71] Shi X Hu Y Zhang B Li W Chen JD Liu F. Ameliorating effects and mechanisms of transcutaneous auricular vagal nerve stimulation on abdominal pain and constipation. JCI Insight. (2021) 6. doi: 10.1172/jci.insight.150052 PMC8410029 34138761

[72] Krames E Mousad DG. Spinal cord stimulation reverses pain and diarrheal episodes of irritable bowel syndrome: a case report. Neuromodulation. (2004) 7:82–8. doi: 10.1111/j.1094-7159.2004.04011.x 22151188

[73] Lind G Winter J Linderoth B Hellström PM. Therapeutic value of spinal cord stimulation in irritable bowel syndrome: a randomized crossover pilot study. Am J Physiol Regul Integr Comp Physiol. (2015) 308:R887–94. doi: 10.1152/ajpregu.00022.2015 25786486

[74] Vitton V Damon H Roman S Nancey S Flourié B Mion F. Transcutaneous posterior tibial nerve stimulation for fecal incontinence in inflammatory bowel disease patients: a therapeutic option? Inflammation Bowel Dis. (2009) 15:402–5. doi: 10.1002/ibd.20774 18972550

[75] Krasaelap A Sood MR Li BUK Unteutsch R Yan K Nugent M. Efficacy of auricular neurostimulation in adolescents with irritable bowel syndrome in a randomized, double-blind trial. Clin Gastroenterol Hepatol. (2020) 18:1987–1994.e2. doi: 10.1016/j.cgh.2019.10.012 31622740

[76] Hu P Sun K Li H Qi X Gong J Zhang Y. Transcutaneous electrical acustimulation improved the quality of life in patients with diarrhea-irritable bowel syndrome. Neuromodulation. (2022) 25:1165–72. doi: 10.1016/j.neurom.2021.10.009 35088760

[77] Li G Jin B Fan Z. Clinical application of transcranial magnetic stimulation for functional bowel disease. Front Med (Lausanne). (2023) 10:1213067. doi: 10.3389/fmed.2023.1213067 37396913 PMC10311555

[78] Pellissier S Bonaz B. The place of stress and emotions in the irritable bowel syndrome. Vitam Horm. (2017) 103:327–54. doi: 10.1016/bs.vh.2016.09.005 28061975

[79] Chang L. The role of stress on physiologic responses and clinical symptoms in irritable bowel syndrome. Gastroenterology. (2011) 140:761–5. doi: 10.1053/j.gastro.2011.01.032 PMC3039211 21256129

[80] Spetalen S Sandvik L Blomhoff S Jacobsen MB. Autonomic function at rest and in response to emotional and rectal stimuli in women with irritable bowel syndrome. Dig Dis Sci. (2008) 53:1652–9. doi: 10.1007/s10620-007-0066-0 17990112

[81] Dinan TG Quigley EM Ahmed SM Scully P O'Brien S O'Mahony L. Hypothalamic-pituitary-gut axis dysregulation in irritable bowel syndrome: plasma cytokines as a potential biomarker? Gastroenterology. (2006) 130:304–11. doi: 10.1053/j.gastro.2005.11.033 16472586

[82] Kennedy PJ Clarke G Quigley EM Groeger JA Dinan TG Cryan JF. Gut memories: towards a cognitive neurobiology of irritable bowel syndrome. Neurosci Biobehav Rev. (2012) 36:310–40. doi: 10.1016/j.neubiorev.2011.07.001 21777613

[83] Labad J Soria V Salvat-Pujol N Segalàs C Real E Urretavizcaya M. Hypothalamic-pituitary-adrenal axis activity in the comorbidity between obsessive-compulsive disorder and major depression. Psychoneuroendocrinology. (2018) 93:20–8. doi: 10.1016/j.psyneuen.2018.04.008 29684711

[84] Waters RP Rivalan M Bangasser DA Deussing JM Ising M Wood SK. Evidence for the role of corticotropin-releasing factor in major depressive disorder. Neurosci Biobehav Rev. (2015) 58:63–78. doi: 10.1016/j.neubiorev.2015.07.011 26271720 PMC4828243

[85] Tache Y Larauche M Yuan PQ Million M. Brain and gut CRF signaling: biological actions and role in the gastrointestinal tract. Curr Mol Pharmacol. (2018) 11:51–71. doi: 10.2174/1874467210666170224095741 28240194 PMC8091865

[86] Chen XF Guo Y Lu XQ Qi L Xu KH Chen Y. Aberrant intraregional brain activity and functional connectivity in patients with diarrhea-predominant irritable bowel syndrome. Front Neurosci. (2021) 15:721822. doi: 10.3389/fnins.2021.721822 34539337 PMC8446353

[87] Liang YF Chen XQ Zhang MT Tang HY Shen GM. Research progress of central and peripheral corticotropin-releasing hormone in irritable bowel syndrome with comorbid dysthymic disorders. Gut Liver. (2024) 18:391–403. doi: 10.5009/gnl220346 37551453 PMC11096901

[88] Raposo-Lima C Morgado P. The role of stress in obsessive-compulsive disorder: A narrative review. Harv Rev Psychiatry. (2020) 28:356–70. doi: 10.1097/HRP.0000000000000274 33027102

[89] Adams TG Kelmendi B Brake CA Gruner P Badour CL Pittenger C. The role of stress in the pathogenesis and maintenance of obsessive-compulsive disorder. Chronic Stress (Thousand Oaks). (2018) 2:1–11. doi: 10.1177/2470547018758043 PMC5841259 29527593

[90] Mar-Barrutia L Ibarrondo O Mar J Real E Segalàs C Bertolín S. Long-term comparative effectiveness of deep brain stimulation in severe obsessive-compulsive disorder. Brain Stimul. (2022) 15:1128–38. doi: 10.1016/j.brs.2022.07.050 35926783

[91] Naesström M Hariz M Strömsten L Bodlund O Blomstedt P. Deep brain stimulation in the bed nucleus of stria terminalis in obsessive-compulsive disorder-1-year follow-up. World Neurosurg. (2021) 149:e794–802. doi: 10.1016/j.wneu.2021.01.097 33540102

[92] Nuttin B Gielen F van Kuyck K Wu H Luyten L Welkenhuysen M. Targeting bed nucleus of the stria terminalis for severe obsessive-compulsive disorder: more unexpected lead placement in obsessive-compulsive disorder than in surgery for movement disorders. World Neurosurg. (2013) 80:S30.e11–6. doi: 10.1016/j.wneu.2012.12.029 23268197

[93] Farrand S Evans AH Mangelsdorf S Loi SM Mocellin R Borham A. Deep brain stimulation for severe treatment-resistant obsessive-compulsive disorder: An open-label case series. Aust N Z J Psychiatry. (2018) 52:699–708. doi: 10.1177/0004867417731819 28965430

[94] Islam L Franzini A Messina G Scarone S Gambini O. Deep brain stimulation of the nucleus accumbens and bed nucleus of stria terminalis for obsessive-compulsive disorder: a case series. World Neurosurg. (2015) 83:657–63. doi: 10.1016/j.wneu.2014.12.024 25527882

[95] Flavin SA Winder DG. Noradrenergic control of the bed nucleus of the stria terminalis in stress and reward. Neuropharmacology. (2013) 70:324–30. doi: 10.1016/j.neuropharm.2013.02.013 PMC3644325 23466330

[96] Fudge JL Haber SN. Bed nucleus of the stria terminalis and extended amygdala inputs to dopamine subpopulations in primates. Neuroscience. (2001) 104:807–27. doi: 10.1016/S0306-4522(01)00112-9 11440812

[97] Crestani CC Alves FH Gomes FV Resstel LB Correa FM Herman JP. Mechanisms in the bed nucleus of the stria terminalis involved in control of autonomic and neuroendocrine functions: a review. Curr Neuropharmacol. (2013) 11:141–59. doi: 10.2174/1570159X11311020002 PMC3637669 23997750

