# Supplementary material for: Deep brain stimulation for obsessive compulsive disorder leads to symptom changes of comorbid irritable bowel syndrome
Source: Front Psychiatry. 2025 Mar 5;16:1545318. doi: 10.3389/fpsyt.2025.1545318 (PMC11919902; doi:10.3389/fpsyt.2025.1545318)
Supplement: Supplementary file 1 [file Table1.docx]

**Supplementary materials:

GSRS-IBS scores**

| **Patient** | **Baseline** | **Before stimulation** | **Optimized stimulation** | **3 months  follow-up** | **6 months  follow-up** | **9 months follow-up** | **12 months follow-up** |
| --- | --- | --- | --- | --- | --- | --- | --- |
| **1** | 50 | * | * | 51 | 43 | 39 | 44 |
| **2** | 15 | 15 | 13 | 13 | 13 | 13 | 13 |
| **3** | 28 | 17 | 27 | 21 | 20 | 17 | 22 |
| **4** | 22 | 23 | * | 16 | 17 | 13 | 17 |
| **5** | 20 | 38 | 30 | 38 | 36 | 29 | 33 |
| **6** | 44 | 34 | 34 | 34 | 42 | 26 | 26 |
| **7** | 33 | 49 | 27 | 24 | 31 | 26 | 23 |
| **8** | 18 | 16 | 18 | 13 | 13 | 17 | 13 |
| **9** | 20 | 15 | 24 | 16 | 19 | -- | -- |

*missing
-- not due yet

 **Y-BOCS scores**

| **Patient** | **YBOCS** | **Baseline** | **Before stimulation** | **Optimized stimulation** | **3 months  follow-up** | **6 months  follow-up** | **9 months follow-up** | **12 months follow-up** |
| --- | --- | --- | --- | --- | --- | --- | --- | --- |
| **1** | Total  Obsessions  Compulsions | 37  20  17 | 35  18  17 | 23  14  9 | 16  9  7 | 15  8  7 | 16  9  7 | 14  7  7 |
| **2** | Total  Obsessions  Compulsions | 33  17  16 | 33  17  16 | 30  15  15 | 26  13  13 | 26  13  13 | 28  14  14 | 28  14  14 |
| **3** | Total  Obsessions  Compulsions | 31  18  13 | 33  19  14 | 12  6  6 | 26  11  15 | 12  6  6 | 10  5  5 | 11  5  6 |
| **4** | Total  Obsessions  Compulsions | 23  20  3 | 26  16  10 | 11  4  7 | 5  0  5 | 5  1  4 | 6  2  4 | 6  1  5 |
| **5** | Total  Obsessions  Compulsions | 34  17  17 | 34  16  18 | 22  11  11 | 24  13  11 | 29  16  13 | 31  17  14 | 30  15  15 |
| **6** | Total  Obsessions  Compulsions | 33  18  15 | 33  18  15 | 16  8  8 | 16  8  8 | 16  9  7 | 15  8  7 | 18  9  9 |
| **7** | Total  Obsessions  Compulsions | 26  8  18 | 29  12  17 | 9  0  9 | 11  3  8 | 15  7  8 | 13  5  8 | 14  7  7 |
| **8** | Total  Obsessions  Compulsions | 36  19  17 | 32  17  15 | 14  8  6 | 10  5  5 | 9  6  3 | 10  5  5 | 11   6  5 |
| **9** | Total  Obsessions  Compulsions | 27  14  13 | 28  16  12 | 15  10  5 | 17  11  6 | 17  11  6 | -- | -- |

*missing
-- not due yet
